# Deciphering aquaporin-4’s influence on perivascular diffusion indices using DTI in rat stroke studies

**DOI:** 10.3389/fnins.2025.1566957

**Published:** 2025-08-05

**Authors:** Jiaqi Tian, Yuwen Zhang, Le Liu, Chaofan Li, Xiaozhu Hao

**Affiliations:** ^1^Department of Radiology, Renji Hospital, School of Medicine, Shanghai Jiao Tong University, Shanghai, China; ^2^College of Health Science and Technology, Shanghai Jiao Tong University School of Medicine, Shanghai, China; ^3^Institute of Science and Technology for Brain-Inspired Intelligence, Fudan University, Shanghai, China; ^4^School of Rehabilitation Science, Shanghai University of Traditional Chinese Medicine, Shanghai, China; ^5^Department of Radiology, Huashan Hospital, Fudan University, Shanghai, China

**Keywords:** cerebral ischemia, glymphatic pathway, DTI, ALPS index, AQP4 polarization

## Abstract

**Background:**

This study aimed to evaluate the dynamic changes of the perivascular space diffusion index (index for diffusivity along the perivascular space, ALPS) and its relationship with aquaporin 4 (AQP4) polarization after cerebral ischemia in rats.

**Methods:**

Rats were subjected to transient middle cerebral artery occlusion (tMCAO) and evaluated at 1, 3, 7, 14, and 28 days post-ischemia using diffusion tensor imaging (DTI), T2-weighted imaging (T2WI), and susceptibility-weighted imaging (SWI). The ALPS index was determined from imaging data, focusing on periventricular and corpus callosum/cingulate regions. Brains were analyzed for AQP4 and glial fibrillary acidic protein (GFAP) via immunofluorescence.

**Results:**

The results showed that ischemic rats displayed reduced ALPS indexes, particularly on the ipsilateral side, with an initial decrease at day 1 and subsequent recovery by days 14 and 28. AQP4 polarization in the non-glial scar area around the infarction followed a similar pattern, demonstrating that there was a concordant trend between the ALPS index and AQP4 polarization status.

**Conclusion:**

In conclusion, the ALPS index can reflect changes in AQP4-mediated glymphatic pathway function, suggesting a significant decline in the hyperacute phase and a notable recovery in the early chronic phase, which may have implications for stroke therapeutic strategies.

## Introduction

Ischemic stroke patients may experience neurological dysfunction (Campbell et al., 2019; Campbell et al., 2021; Fukuta et al., 2022; Schaechter et al., 2009; Alawieh et al., 2018), highlighting the urgent need to develop new therapies that can reduce brain injury and promote brain repair after stroke. The glymphatic pathway has been proposed as a research hotspot in this field in recent years. Researchers pointed out that there is a significant correlation between glymphatic function and the prognosis of sensorimotor prognosis after stroke. The glymphatic pathway is a perivascular network in the brain responsible for the exchange of cerebrospinal fluid and interstitial fluid. It consists of three main structures: the cerebrospinal fluid inflow channel around the artery, the interstitial fluid outflow channel around the vein, and the astrocyte exchange channel connecting the two channels (Iliff et al., 2012; Benveniste et al., 2019). Cerebrospinal fluid flows into the brain through the perivascular space of the large pia mater artery at the beginning of the glymphatic pathway and then enters the brain parenchyma via the perivascular space along the vascular branches (Iliff et al., 2012; Aspelund et al., 2015; Ma et al., 2017; Hannocks et al., 2018; Iliff et al., 2013; Pizzo et al., 2018). In the brain parenchyma, cerebrospinal fluid is mixed with interstitial fluid, flowing to the perivenous space via global convection (Iliff et al., 2012; Kress et al., 2014), and is finally excreted out of the brain through meningeal lymphatic vessels and arachnoid granules (Holter et al., 2017; Smith et al., 2017). Cerebrospinal fluid enters and exits from the brain parenchyma through the aquaporin 4 (AQP4), which is expressed in the foot processes of astrocytes in the perivascular space of arteries and veins.

The traditional method to evaluate the glymphatic function is dynamic contrast-enhanced magnetic resonance imaging (DCE-MRI) with intrathecal or cisternal injection of gadolinium-based contrast agent. Cerebrospinal fluid drainage and clearance are visualized through T1 signal enhancement changes caused by gadolinium paramagnetism. However, intrathecal injection is typically limited to patients who need lumbar puncture for other purposes and cannot be widely used as a tool for disease screening, and cisternography is currently limited to animal research. Taoka et al. (2017) proposed a method of diffusion tensor image analysis along the perivascular space (DTI-ALPS), which is a non-invasive method based on diffusion tensor imaging to evaluate the glymphatic function in subjects. In the brain parenchyma, water molecules move along the perivascular space formed by arteries and veins. At the level of lateral ventricle body, the medullary vein travels in the left–right direction, while the projecting fiber travels in the cephalocaudal direction and the association fiber in the ventrodorsal direction. Diffusion change of water molecules along the horizontal axis in the projection fiber and the association fiber can be attributed to changes in diffusion via the perivascular space of the medullary vein. The ALPS index, which is the ratio of axial diffusion to radial diffusion of projection and association fiber, was used to evaluate the activity of glymphatic pathway (Taoka et al., 2017). Studies have confirmed the significant correlation between DTI-ALPS and DCE-MRI, indicating that the ALPS index can reflect the function of glymphatic pathway (Zhang et al., 2021).

To date, DTI-ALPS has been used in several diseases, including Alzheimer’s disease, hydrocephalus, Parkinson’s disease, stroke, and others (Taoka et al., 2017; Yang et al., 2022; Bae et al., 2021; Ma et al., 2021; Toh and Siow, 2021). In stroke patients, the ALPS index is significantly decreased, suggesting the impairment of glymphatic pathway (Toh and Siow, 2021; Qin et al., 2023; Wang et al., 2017). Brain glymphatic activity not only contributes to the pathological process of ischemic stroke but also mediates its recovery. Researchers have pointed out that changes in glymphatic activity after ischemic stroke may be related to time since stroke onset, which needs to be elucidated by longitudinal studies (Qin et al., 2023). Thus, the dynamic changes of glymphatic function after ischemic stroke and its mechanism need to be further studied.

The glymphatic pathway is an integrated system affected by many factors including cardiac impulse, arteriopulmus, consciousness, perivascular space size, cerebrospinal fluid production rate, expression and localization of AQP4, and these factors play different roles in various diseases (Arbel-Ornath et al., 2013; Wang et al., 2023; Gaberel et al., 2014; Ji et al., 2021; Lv et al., 2021; Li et al., 2022). Astrocytic endfeet, together with AQP4, cover most of the brain microvessels to participate in the structure of perivascular space and blood–brain barrier (BBB) and thus form a key part of the glymphatic pathway. Studies showed that the infarction manifested the diffuse elevation of AQP4 expression in the early stage after cerebral ischemia, which is a main cause of cellular edema. Then, in the chronic stage after stroke, the global glymphatic function returned to normal as AQP4 expression returned to the baseline levels (Wang et al., 2012), while focal glymphatic impairment persisted (Wang et al., 2017). Previous studies have also pointed out that, in the late stage after cerebral ischemia, waste solutes from the necrotic core could leak across the glial scar and be removed by the AQP4-mediated glymphatic pathway in the adjacent tissue (Zbesko et al., 2018). Therefore, as an important part of the glymphatic pathway, AQP4 might be a dominant factor in glymphatic function changes (Vella et al., 2015). The calculation of ALPS index in the human brain is based on a specific plane of the lateral ventricle body, and this method cannot be directly applied to rats. It is not yet clear whether changes in the ALPS index can reflect alterations in AQP4 polarization.

Therefore, our study primarily focuses on two issues: 1. The dynamic changes of ALPS index after cerebral ischemia; 2. The spatial and temporal characteristics of AQP4 polarization following cerebral ischemia, and its relationship with the ALPS index. Resolving these questions would provide a theoretical basis for developing therapeutic targets to protect the glymphatic pathway and promote the use of DTI-ALPS as an *in vivo* imaging tool for evaluating glymphatic pathway function. The purpose of this study is to explore the dynamic changes in the ALPS index in ischemic rats, as well as the temporal and spatial relationships between the ALPS index and AQP4 polarization.

## Methods

### Animals

A total of 25 Sprague–Dawley rats (260–270 g, 8 weeks old) provided by the Shanghai Experimental Animal Center of Fudan University (license No. SCXK (Hu) 2014–0004) were used in this study. This study was performed in accordance with the guidelines outlined by the institutional Animal Care and Use Committee of Fudan University (Approval No. 2018021165) in February 2018.

### Study design

**Figure 1:**
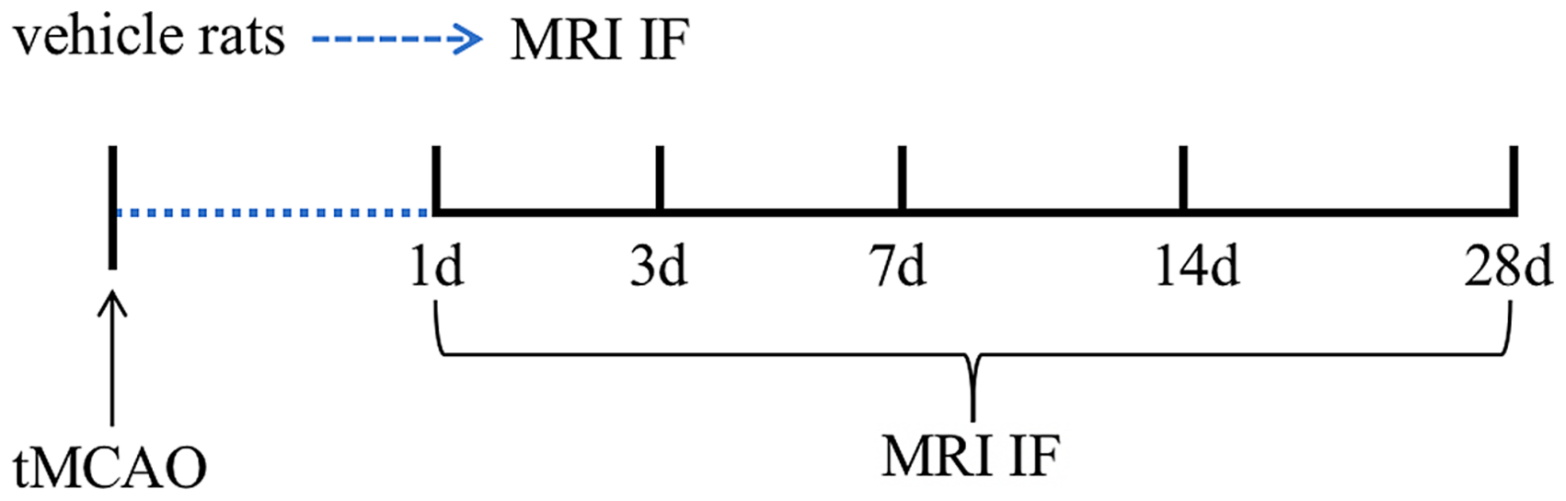
Experimental procedure.

Rats were randomly divided into the vehicle group and the operation group. Rats in the operation group were treated with transient middle cerebral artery occlusion (tMCAO), while rats in the vehicle group underwent the same procedure except for inserting the filament. Ischemic rats were randomly divided into five subgroups. Rats in the vehicle group and subgroup rats at 1 day, 3 days, 7 days, 14 days, and 28 days were, respectively, subjected to MRI scanning (Figure 1). After MRI scanning on day 1, infarct lesions that were too small and did not appear in the cortex and striatum were excluded. To ensure that the number of rats for pathological sampling at each time point is 5, due to mortality, the number of rats for MRI scanning at each time point exceeds the number for pathological sampling. A total of 39 rats were used in this experiment.

### Stroke model

The stroke model was established as we described previously (Hao et al., 2016). Briefly, the rats were first anesthetized with 2% isoflurane (Panion & Biotevh Inc., Taipei, China) (30% O_2_ and 70% N_2_O) until the tail reflex disappeared, and the concentration of isoflurane was maintained at 1–2% during the operation. To generate the tMCAO model, we inserted a filament via the left internal carotid artery to reach the origin of the middle cerebral artery, with the blood flow volume decreased to more than 80% of that before operation. After 90 min, the filament was slowly removed, and the blood flow volume returned to more than 80% of that before the operation.

### Magnetic resonance imaging

Image data were acquired using an 11.7T MRI scanner (Bruker, Ettlingen, Germany) with a 4-channel cryogenic phased array mouse head coil. In addition, the MRI sequences and parameters were as follows: (1) T2-weighted imaging (T2W): repetition time = 4,200 ms, echo time = 25 ms, visual field = 30 mm × 30 mm, slice thickness = 0.5 mm, matrix = 256 × 256; (2) susceptibility weighted imaging (SWI): repetition time = 56 ms, echo time = 32 mm, visual field = 30 mm × 30 mm, layer thickness = 0.5 mm, matrix = 256 mm; (3) diffusion tensor imaging (DTI): gradient direction is 30 directions, selected directions are 0, 1,000 s/mm2, repetition time = 3,000 ms, echo time = 26.26 ms, visual field = 30 mm × 30 mm, layer thickness = 0.5 mm, matrix = 100×100 mm.

### Immunofluorescence labeling

Rats were sacrificed by exposure to carbon dioxide at a continuously increasing concentration until cardiac arrest appeared. After thoracotomy, the rats were perfused with 0.9% saline solution via the left ventricle until the effluent liquid from the auricula dextra was clear. The removed brains were post-fixed in 4% paraformaldehyde solution for 24 h and were consecutively dehydrated with 20 and 30% sucrose solution for 3 days. Coronal brain sections (5 μm) were obtained at approximately 0.48 mm relative to the bregma. For immunofluorescence, free-floating sections were washed with PBS (PH 7.4), blocked in 10% normal donkey serum, and incubated with primary antibodies overnight. The primary antibodies used were as follows: (1) rabbit anti-AQP4 (1:200, Millipore, USA); (2) mouse anti-glial fibrillary acidic protein (GFAP) (1:300, GeneTex, USA), a marker of astrocytes. Then, the sections were washed with PBS and then labeled with secondary antibodies for 2 h. The secondary antibodies used were Alexa Fluor 488- or 568-conjugated donkey anti-rabbit and anti-mouse (1:200, Life Technologies, Carlsbad, USA). Finally, the sections were washed with PBS, counterstained with 4′,6-diamidino-2-phenylindole (1,1,000, Sigma-Aldrich, USA), and cover-slipped with mounting medium.

## Image processing and analysis

### MRI

#### Regions of interest

**Figure 2:**
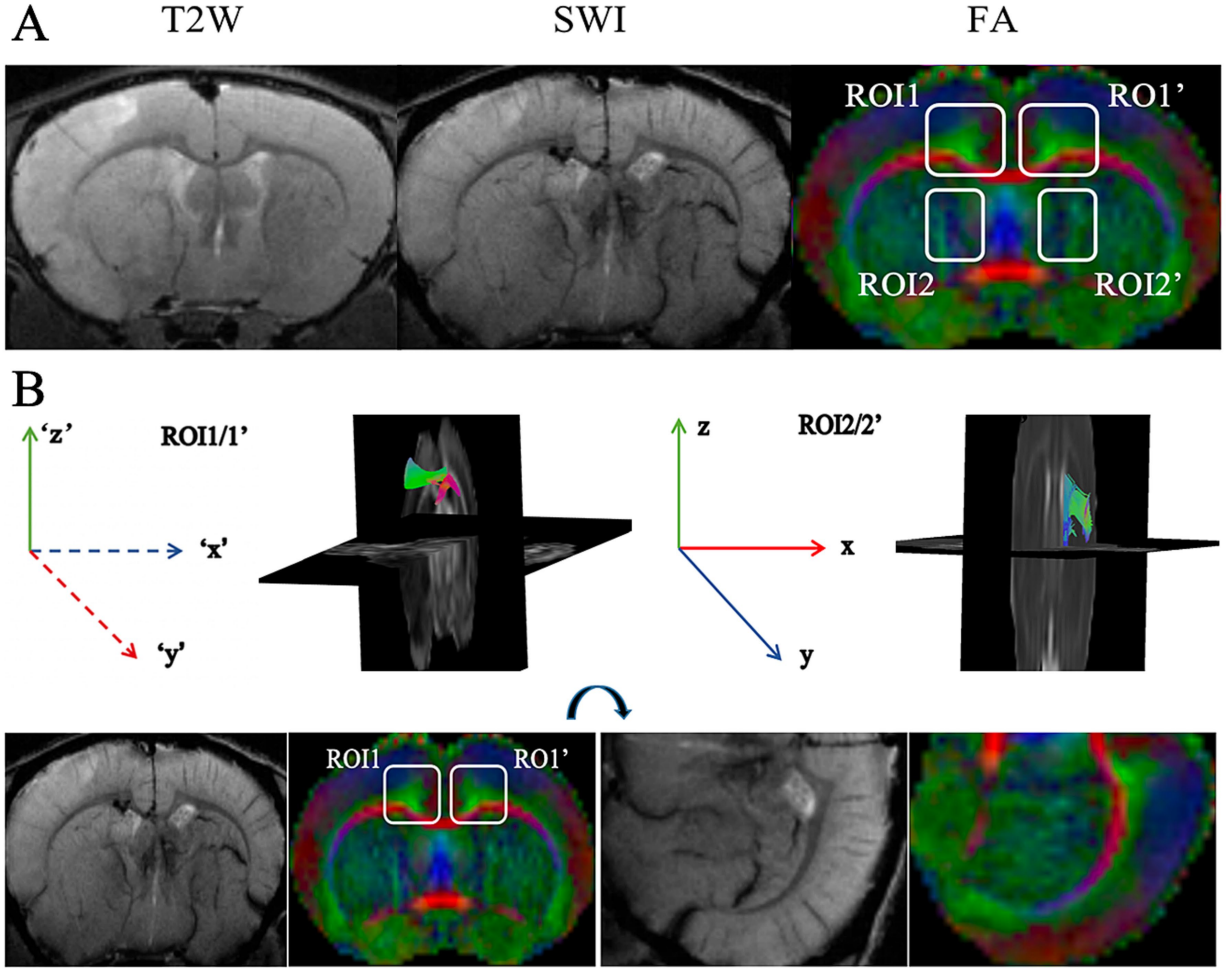
**(A)** Illustration of ROIs. T2WI shows hyperintensity of the ischemic lesion located in the left cortex and striatum. SWI shows the direction of veins. The FA map shows ROIs and the direction of axon bundles. ROIs are placed in the bilateral corpus callosum/cingulate gyrus (corpus callosum, red; cingulate gyrus, green) and peri-lateral ventricular area (ventrodorsal, blue; cephalocaudal, green). **(B)** The direction illustration of fiber bundles within ROIs. For ROI2 and ROI2’, ventrodorsal fibers run along the *y*-axis and cephalocaudal fibers run along the *z*-axis. For ROI1 and ROI1’, in the flipped images, the corpus callosum runs along the ‘*y*’-axis and the cingulate gyrus runs along the ‘*z*’-axis.

According to the calculation principle of the ALPS index, fibers and veins should be perpendicular to each other. In the previous study, the region of interest (ROI) was placed beside the lateral ventricle body, where the projection fiber, association fiber, and medullary veins are perpendicular to each other. In this study, by observing the course of fibers in the fractional anisotropy (FA) map and veins in SWI, we found two targeted ROIs (Figure 2A). One region is beside the lateral ventricle body, containing three structures that are perpendicular to each other, consistent with the previous study. In this region, medullary veins run in left–right direction (*x*-axis), while projection fibers run in cephalocaudal direction (y-axis) and association fibers run in ventrodorsal direction (*z*-axis). Another potential region is the intersection of the corpus callosum and cingulate gyrus. Specifically, in this region, medullary veins run in a ventrodorsal direction, while the corpus callosum runs in a left–right direction, and the cingulate gyrus runs in a cephalocaudal direction. To fit in with the ALPS formula, we flipped the FA and SWI images 90 degrees. Accordingly, in the flipped images, medullary veins run in left–right direction (‘x’-axis), while the corpus callosum runs in ventrodorsal direction (‘y’-axis) and cingulate gyrus runs in the cephalocaudal direction (‘z’-axis) (Figure 2B). We proposed the ROI of corpus callosum/cingulate gyrus for the first time to exploit the calculation area of ALPS. Therefore, two sites and their mirror positions were selected as ROIs: (1) ROI1: corpus callosum/cingulate gyrus in the ipsilateral side; (2) ROI1’: corpus callosum/cingulate gyrus in the contralateral side; (3) ROI2: area beside the ipsilateral ventricle body; (4) ROI2’: area beside the contralateral ventricle body. In the color FA map, red indicates cephalocaudal direction (x and ‘y’-axis), while green indicates ventrodorsal direction (z and ‘z’-axis), and blue indicates left–right direction (y and ‘x’-axis). For ALPS index, formula in ROI1 and ROI1’ is expressed as ALPS _(ROI1, ROI1’)_ = (Dx_cingulate gyrus_ + Dx_corpus callosum_)/(Dy_cingulate gyrus_+ Dz_corpus callosum_), and formula in ROI2 and ROI2’ is expressed as ALPS _(ROI2, ROI2’)_ = (Dx_cephalocaudal_ + Dx_ventrodorsal_)/(Dy_cephalocaudal_ + Dz_ventrodorsal_).

#### DTI processing

All DTI images in raw Bruker ParaVision data format were converted to NIFIT using the Python tool “bruker2nifti,” and the voxel size was extended by zooming ×10 to be consistent with the template. Then they were identically preprocessed using the functional magnetic resonance imaging of the brain (FMRIB) software library tool (FSL, version 6.0.4, Oxford, UK). The initial DTI images were corrected for motion and eddy currents by eddy (a tool in the FSL suite). Prior to calculating the b matrices, the b-vector was recalculated after motion correction to account for the rotational component introduced by eddy. A brain mask was defined for each subject on the averaged B0 images using the rodent brain extraction toolbox “3D PCNN.” The Turone Mouse Brain Template and Atlas (TMBTA), which is a complete set of *ex vivo* templates, tissues priors, as well as an anatomical atlas of the mouse brain, was employed as the standard space for subsequent analyses. To align the individual subject with the template, spatial normalization was accomplished through SPM with a non-linear registration algorithm. Finally, DTFIT of FSL was used to independently fit diffusion tensors to brain space. The output of DTIFIT yielded voxel-wise maps of FA for each rat.

**Figure 3:**
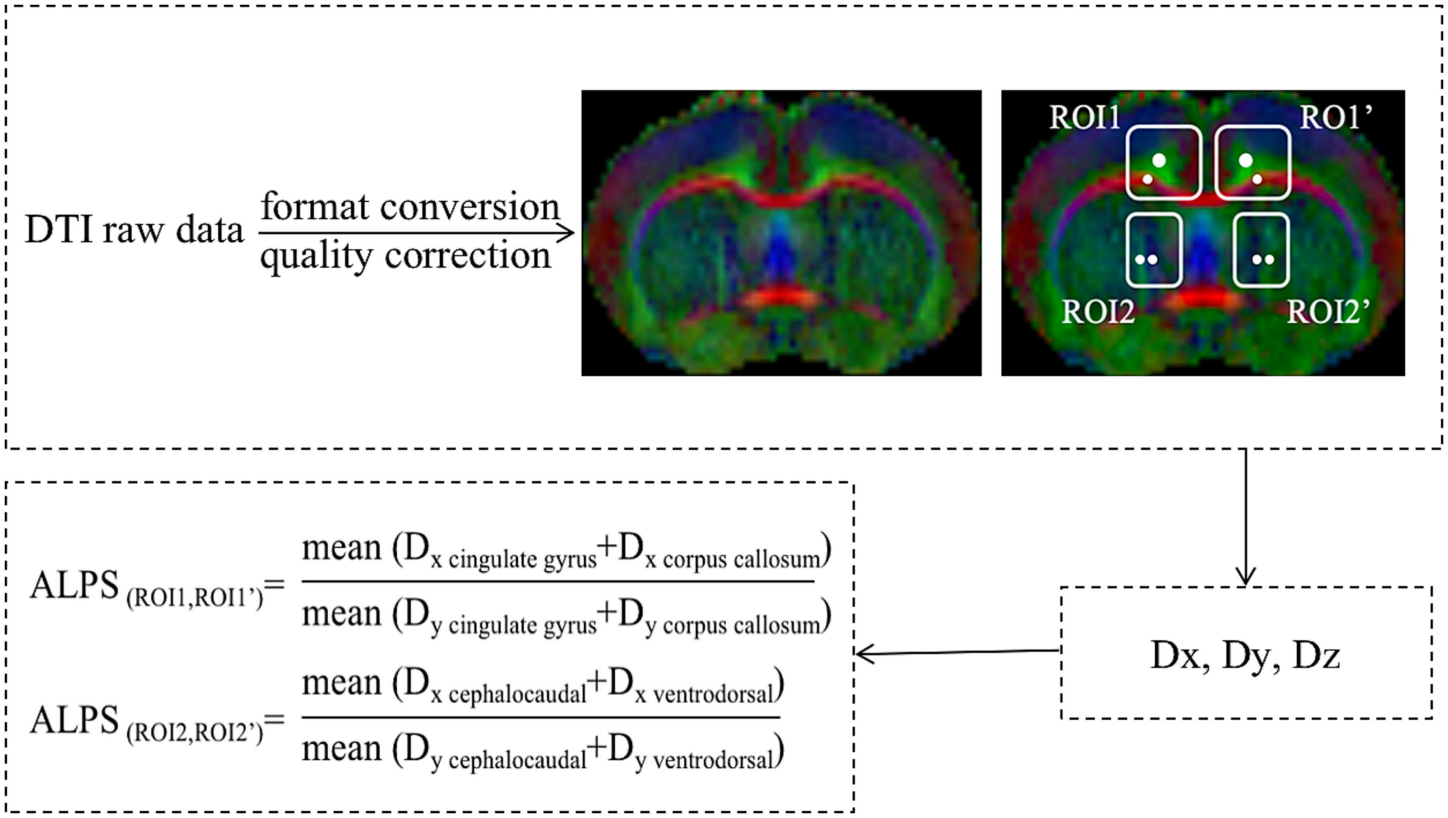
Flowcharts for ALPS index calculation.

The processed images of FA were opened by ITK-SNAP software, and measuring positions were placed on fibers in the four selected ROIs: ROI1 (ipsilateral corpus callosum and cingulate gyrus), ROI1’ (contralateral corpus callosum and cingulate gyrus), ROI2 (cephalocaudal fiber and ventrodorsal fiber beside the ipsilateral ventricle body) and ROI2’ (cephalocaudal fiber and ventrodorsal fiber beside the contralateral ventricle body). Finally, Dx, Dy, and Dz values of these fibers are extracted and brought into the formula to calculate ALPS indexes (Figure 3).

### Immunofluorescence analysis

#### Regions of interest

In response to ischemia, astrocytes undergo morphological and functional plasticity with temporal and spatial features. In the chronic stage of stroke, astrocytic gliosis occurs around the infarction and presents a gradient decreasing trend. Close to the lesion core, a glial scar was constructed to separate the necrotic and the healthy brain tissue. Astrocytes in this area play the role of a physical barrier, but have thoroughly lost their physiological functions. While in the transition zone between glial scar and healthy tissue, astrocytes exhibit mild hypertrophy with impaired but retained function. Thus, in this study, ROIs were selected in the transition zone beside the infarction as well as mirror areas to evaluate the impairment and recovery of the glymphatic pathway.

#### Calculation of AQP4 intensity and polarity

GFAP/AQP4 confocal images of the selected ROIs were obtained and imported into Image J software. Firstly, the AQP4 intensity of each image was calculated. Next, for analysis of AQP4 polarity, each image is divided into a low stringency threshold and a high stringency threshold. The former defined the overall area occupied by AQP4, while the latter defined the designated area occupied by AQP4 localized to perivascular endfeet. The ratio of a low stringency threshold to the high stringency threshold defines the AQP4 polarity. A higher AQP4 polarity indicates more centralized expression of AQP4 in perivascular endfeet, whereas a lower AQP4 polarity suggests more diffusive expression of AQP4 in brain tissue.

### Statistical analysis

All statistics were performed using GraphPad Prism (version 10.0). The Kolmogorov–Smirnov test was employed to ascertain the normality of the data distributions. One-way analysis of variance (ANOVA) was performed for multiple group comparisons with Tukey’s *post-hoc* test for each of the two groups. Data were presented as mean ± SD. The results were considered statistically significant if the *p*-value was lower than 0.05.

## Results

### Dynamic changes of T2W images of infarction over time

**Figure 4:**
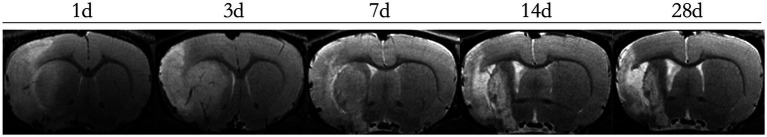
Dynamic changes of T2WI at different times. The infarction appeared as T2 hyperintensity with swelling of the ipsilateral hemisphere 1 and 3 days after stroke, and developed into malacia 14 and 28 days after stroke.

The dynamic changes of the infarction were assessed by T2W images (Figure 4). Lesions were observed in the left cortex and striatum. Infarction-related edema occurred in the acute stage (day 1, day 3), appearing as T2 hyperintensity and causing swelling of the ipsilateral hemisphere as well as compression of the contralateral hemisphere. Then, in the subacute stage (day 7), attenuating edema and increasing cell proliferation led to pseudo-normalization of the T2 signal. By the chronic stage (day 14, day 28), the lesion core had gradually developed into malacia, manifesting as abnormal T2 hyperintensity almost equal to cerebrospinal fluid. Concurrently, cerebral necrosis led to atrophy of the ipsilateral hemisphere.

### Dynamic changes of the ALPS indexes over time

**Figure 5:**
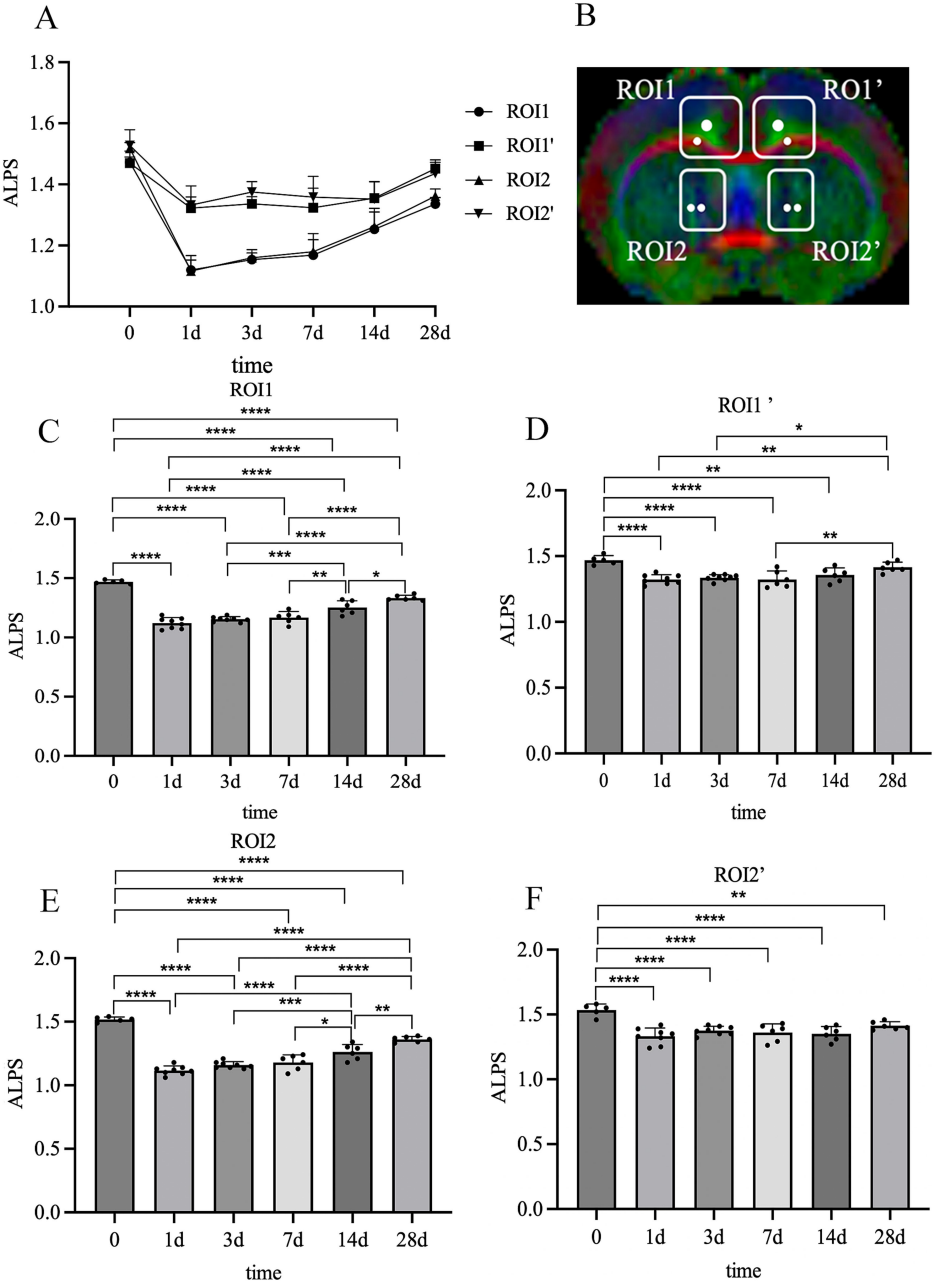
Dynamic changes of ALPS indexes at different times. **(A)** ALPS index trends. ALPS indexes significantly decreased 1 day after stroke and then gradually increased at later timepoints **(B)** Diagram of ROIs for ALPS indexes. **(C)** Dynamic changes of ALPS indexes in ROI1. **(D)** Dynamic changes of ALPS indexes in ROI1’. **(E)** Dynamic changes of ALPS indexes in ROI2. **(F)** Dynamic changes of ALPS indexes in ROI2’. ^*^p < 0.05, ^**^p < 0.01, ^***^p < 0.001, ^****^p < 0.001.

The ALPS indexes in four ROIs showed a consistent temporal evolution after cerebral ischemia (Figures 5A,B). Specifically, the ALPS indexes of both the ipsilateral and contralateral sides in ischemic rats were significantly decreased during the superacute stage (day 1) compared to vehicle rats (^****^p < 0.001), with a more pronounced decrease on the ipsilateral side. Subsequently, ALPS indexes increased slightly during the acute and subacute stage (day 3, day 7), though without statistical significance. By the early chronic stage (day 14) and late chronic stage (day 28), ALPS indexes had significantly increased (Figures 5C–F). Specifically, the ipsilateral ALPS index in the corpus callosum/cingulate gyrus increased significantly at day14 and day 28 compared to each previous timepoint (14 d vs. 1 d, ^****^p < 0.01; 14 d vs. 3 d, ^***^p < 0.01; 14 d vs. 7 d, ^**^p < 0.05; 28 d vs. 1 d, ^****^p < 0.01; 28 d vs. 3 d, ^****^p < 0.01; 28 d vs. 7 d, ^****^p < 0.01; 28 d vs. 14 d, ^*^p < 0.05). The contralateral ALPS index in the corpus callosum/cingulate gyrus increased significantly at day 28 compared to days 1, 3, and 7 (28 d vs. 1 d, ^**^p < 0.01; 28 d vs. 3 d, ^*^p < 0.05; 28 d vs. 7 d, ^**^p < 0.01). Similarly, the ipsilateral ALPS index in the area adjacent to the ventricle body increased significantly at day14 and day 28 (14 d vs. 1 d, ^****^p < 0.01; 14 d vs. 3 d, ^***^p < 0.01; 14 d vs. 7 d, ^*^p < 0.05; 28 d vs. 1 d, ^****^p < 0.001; 28 d vs. 3 d, ^****^p < 0.01; 28 d vs. 7 d, ^****^p < 0.01; 28 d vs. 14 d, ^**^p < 0.05). Notably, during the chronic stage, the ALPS indexes in ischemic rats remained lower than those in vehicle rats.

### Dynamic changes of AQP4 polarization over time

**Figure 6:**
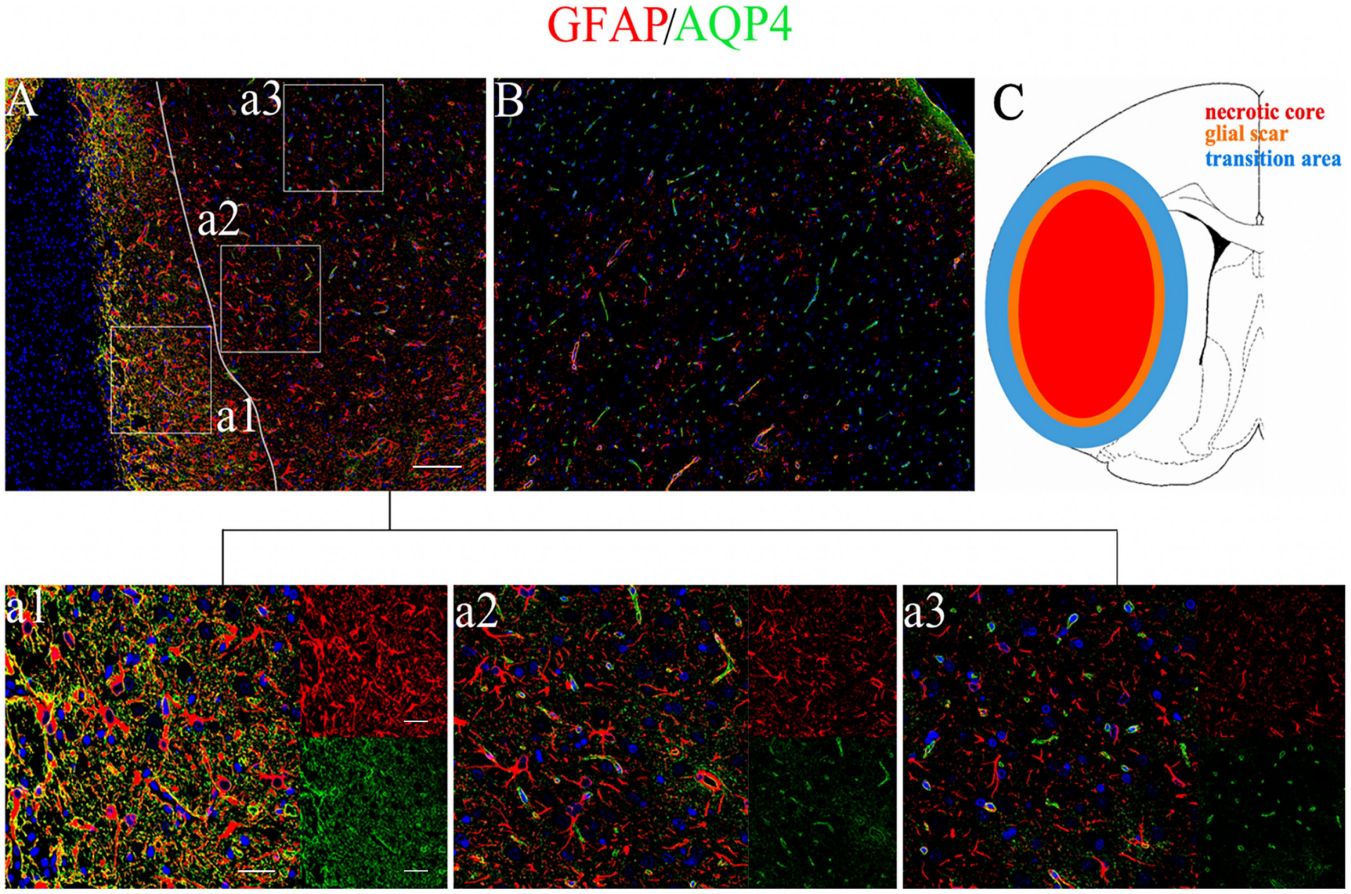
Astrocyte reaction and AQP4 polarity after stroke. **(A)** Immunofluorescence of GFAP/AQP4 in the cortical peri-lesion (28 days after stroke). Scale bar = 100 μm (×10 magnification). The region left to the white line shows the glial scar area **(a1)**. The region right to the white line shows the transition area **(a2)** and the normal area **(a3)**. Scale bar = 20 and 50 μm (×40 and ×20 magnification). **(B)** Immunofluorescence of GFAP/AQP4 in the contralateral cortex. **(C)** Diagram of the necrotic core (red), glial scar (orange), and transition area (blue).

To elucidate the relationship between AQP4 pathology and ALPS index, we further analyzed the AQP4 intensity and polarization. We found that astrocytes and AQP4 exhibit temporal and spatial features in response to ischemic insult. In the area adjacent to the lesion core, astrocytes underwent significant proliferation, eventually forming the glial scar, characterized by abnormal and diffuse AQP4 expression (Figures 6A,a1). Astrocytes in the glial scar function as a physical barrier but lose their physiological functions. Adjacent to the glial scar, astrocytes exhibited mild hypertrophy and proliferation, accompanied by moderately increased expression of AQP4. Unlike the glial scar, astrocytic endfeet and AQP4 retained their structure in this transition area (Figures 6A,a2), suggesting potential plasticity. Further from the infarction, astrocytes and AQP4 exhibited features identical to the contralateral side (Figures 6A,a3,B). Based on the spatiotemporal features of astrocytic plasticity and AQP4 polarity, the lesion area in the chronic stage could be classified into necrotic core, glial scar, and transition area (Figure 6C).

**Figure 7:**
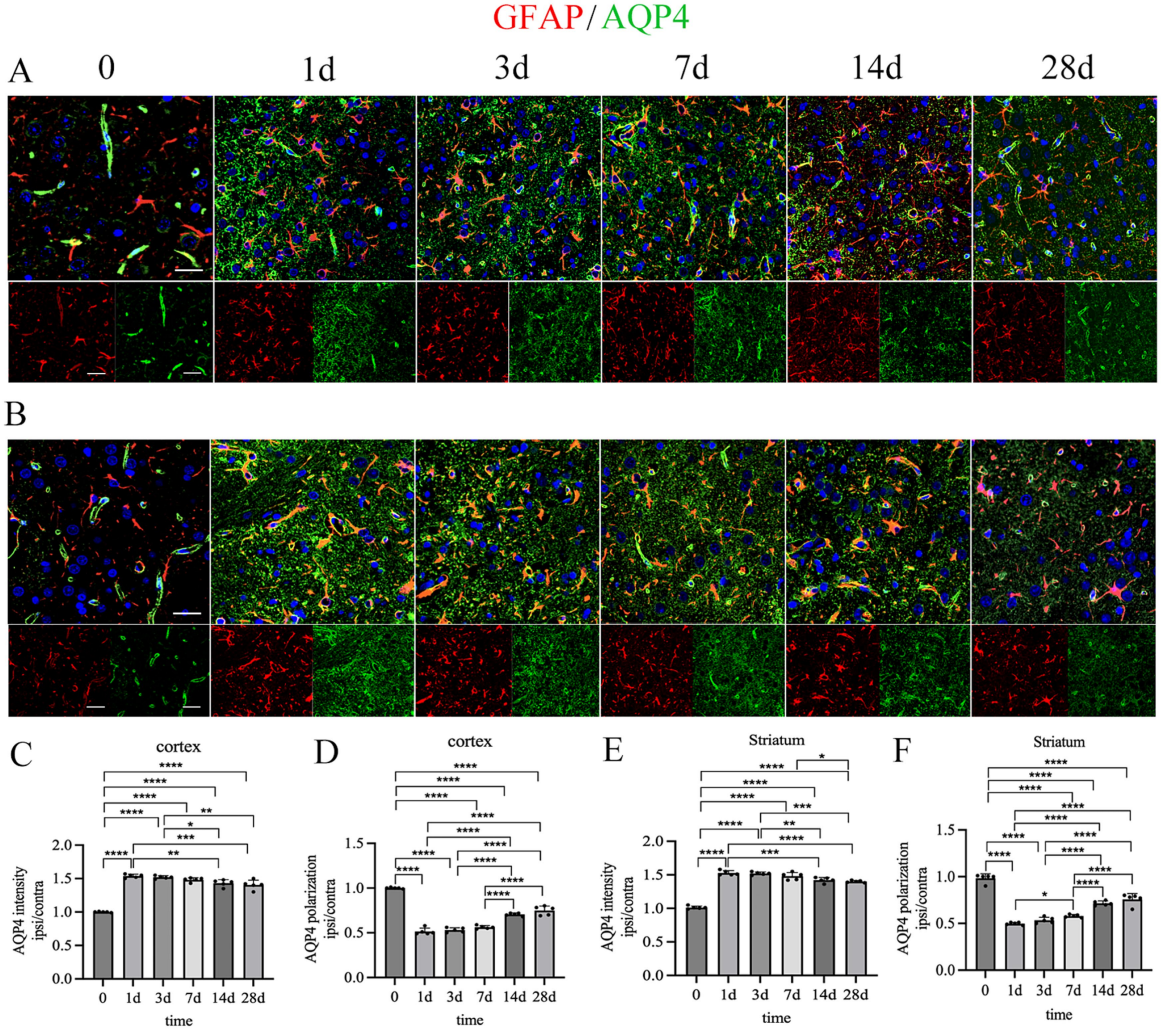
Dynamic changes of AQP4 polarization over time. **(A)** Dynamic changes of AQP4 polarization over time. **(B)** Dynamic changes of AQP4 polarization over time. Scale bar = 20 and 50 μm (×40 and ×20 magnification). Statistical analysis of cortical AQP4 intensity **(C)**, cortical AQP4 polarization **(D)**, striatal AQP4 intensity **(E)** and striatal AQP4 polarization. Both cortical and striatal AQP4 intensity significantly increased at all timepoints, with significant decreases at 14 and 28 days after stroke. Both cortical and striatal AQP4 polarization significantly decreased at all timepoints, with significant increases at 14 and 28 days after stroke. ^*^p < 0.05, ^**^p < 0.01, ^***^p < 0.001, ^****^p < 0.0001.

Therefore, for assessing AQP4 polarization, we placed ROIs in the transition area adjacent to the cortical and striatal infarction. Results showed elevated AQP4 expression in these ipsilateral regions compared to vehicle rats at all timepoints (^****^p < 0.0001 for all comparisons). In ischemic rats, AQP4 intensity showed a gradually decreasing trend, with significant decreases during the chronic stage (cortex, 14 d vs. 1 d, ^**^p < 0.01; 14 d vs. 3 d, ^*^p < 0.05; 28 d vs. 1 d, ^***^p < 0.001; striatum, 14 d vs. 1 d, ^***^p < 0.001; 14 d vs. 3 d, ^**^p < 0.01; 28 d vs. 1 d, ^****^p < 0.001; 28 d vs. 3 d, ^***^p < 0.001) (Figures 7A–C,E). AQP4 polarization decreased in ischemic rats at all timepoints compared to vehicle rats (^****^p < 0.0001 of all comparisons). In ischemic rats, AQP4 polarization showed a gradually increasing trend, with a slightly increase at day 7 (striatum, 7 d vs. 1 d, ^*^p < 0.05) and significantly increases at day14 (both cortex and striatum, 14 d vs. 1 d, ^****^p < 0.0001; 14 d vs. 3 d, ^****^p < 0.0001; 14 d vs. 7 d, ^****^p < 0.0001) and day 28 (both cortex and striatum, 28 d vs. 1 d, ^****^p < 0.0001; 28 d vs. 3 d, ^****^p < 0.0001; 14 d vs. 7 d, ^****^p < 0.0001) (Figures 7A,B,D,F).

## Discussion

In this study, T2W images revealed dynamic changes of the infarction. Ischemic lesions show hyperintensity on T2WI, reflecting edema and cell density. At the superacute stage, cellular toxic edema caused swelling of the ipsilateral hemisphere. At the acute stage, subsequent vasogenic edema exacerbated the hemispheric swelling. With edema attenuation and increased proliferation of inflammatory cells, the infarction exhibited pseudo-normalization of T2 intensity during the subacute stage. Finally, edema and maladaptive responses led to energy deprivation and cell rupture, explaining the development of infarction necrosis. Thus, during the chronic stage, the lesion evolved into cystic encephalomalacia with T2 hyperintensity similar to cerebrospinal fluid.

Then, we monitored *in vivo* functional changes of the glymphatic pathway using the DTI-ALPS method. Previous studies placed ROIs in projection and association fibers adjacent to the lateral ventricle body. In this study, we identified an additional ROI conforming to the calculation principle: the intersection of corpus callosum and cingulate gyrus. In this area, three structures, including the corpus callosum, the cingulate gyrus, and the medullary veins of the cortex, are oriented perpendicular to each other. For ALPS index calculation, image planes were virtually rotated 90 degrees to align with the ALPS coordinate system. The results showed that ALPS indexes in the bilateral corpus callosum/cingulate gyrus followed the same trend as those in peri-ventricular regions. Both ipsilateral and contralateral ALPS indexes significantly decreased after the ischemic insult, gradually recovering over time. Significant increases in ALPS indexes emerged during the early chronic stage after cerebral ischemia. The results indicated that ALPS indexes reflect overall glymphatic function changes after stroke.

To evaluate whether ALPS indexes reflect AQP4 polarization, we analyzed its dynamic changes post-ischemia. Immunofluorescence confirmed liquefactive necrosis in chronic-stage infarcts, consistent with T2WI findings. The encephalomalacia focus was delineated by a glial scar formed by reactive astrocytes. In glial scar regions, reactive astrocytes function as a physical barrier but lack physiological functions. While in the transition area beside the glial scar, reactive astrocytes exhibited hypertrophy while maintaining their morphology, indicating plasticity potential. AQP4 expression was markedly elevated and diffusely distributed in the glial scar, whereas in the transition zone, it was moderately increased and predominantly retained perivascular localization. Thus, for AQP4 analysis, we positioned measurement sites in transition zones surrounding cortical and striatal infarctions. In these regions, AQP4 polarization decreased significantly after ischemic insult but gradually recovered over time. By the early chronic stage, AQP4 polarization increased significantly compared to earlier timepoints.

Thus, our study established the temporal profile of ALPS indexes, demonstrating *in vivo* dynamic changes of glymphatic function, and clarified the relationship between ALPS indexes and AQP4 polarization in peri-infarct transition zones. Prior research reported a transient suppression of the global glymphatic function in microinfarction rats with injection of dextran into the cisterna magna, and such suppression is resolved within 2 weeks (Wang et al., 2017). For astrocytes and AQP4, the reaction of astrocytes was apparent but attenuated 28 days after injury, and AQP4 polarization was significantly reduced at 3 days and successively increased at 14 days and 28 days after stroke (Wang et al., 2012). Our data confirm global glymphatic impairment upon ischemia and subsequent chronic-phase recovery using *in vivo* DTI-ALPS. AQP4 polarization dynamics in transition zones aligned with prior reports. Thus, ischemic injury induces global glymphatic dysfunction that is suppressed acutely but recovers chronically, with the extent of recovery dependent on injury severity. In our tMCAO model, glymphatic function partially recovered but remained subnormal chronically, whereas microinfarction models showed full normalization in a previous study. Critically, the impairment and recovery of the glymphatic pathway correlated with decreased and increased AQP4 polarization, respectively.

Furthermore, we delineated spatially distinct AQP4 polarization patterns relative to the infarct. Based on vascular occlusion extent, infarcts are classified into two types: incomplete microinfarction and cavitated infarction, represented by MCAO models (Wang et al., 2012). The former type is characteristic of diffusive astrogliosis but no defined glial scar, and the reactive astrocyte function was impaired but could recover at the chronic stage after stroke (Wang et al., 2012). Conversely, cavitated infarcts undergo rapid core necrosis progressing to liquefaction, while peri-lesional astrocytes proliferate, forming a stable glial scar (Wang and Parpura, 2016; Nguyen et al., 2016). AQP4 expression is immediately and significantly increased in the lesion core, and then AQP4 expression in the lesion core was gradually decomposed within several hours after stroke, and only expressed in the peri-lesion (Wang and Parpura, 2016; Ribeiro Mde et al., 2006; Huang et al., 2013). In the area of glial scar, AQP4 was diffusively distributed with the gradual increase of expression. However, in the transition area adjacent to the glial scar, reactive astrocytes were mildly proliferated and the AQP4 was moderately expressed. In this area, a part of AQP4 still retained the perivascular localization, and the AQP4 polarization was decreased at the superacute and acute stage, while it was reversely increased at the chronic stage. Previous studies also revealed that the spatial distribution of AQP4 is closely associated with its function (Yang et al., 2011), and AQP4 in the surrounding parenchyma adjacent to the glial scar is important to the waste solute clearance (Zbesko et al., 2018). Thus, for the MCAO model, AQP4 in the transition area of the peri-lesion possesses the potential for plasticity and contributes to the recovery of the whole glymphatic function after stroke.

Previously, it was generally believed that the brain lacked a conventional glymphatic system until the concept of glymphatic pathway in the brain was proposed, and researchers found that the glymphatic pathway removes cellular waste from the brain by exchanging the cerebrospinal fluid and interstitial fluid (Iliff et al., 2012). The function of the glymphatic pathway mainly depends on AQP4, which acts as a promoter to drive the transport of water along the perivascular space (Rasmussen et al., 2018; Buffoli, 2010; Mestre et al., 2018). After ischemic stroke, the impairment of the glymphatic pathway results in the retention of waste solute in the lesion core and leads to post-stroke dementia (Back et al., 2020; Rangroo Thrane et al., 2013; Achariyar et al., 2016). Our study establishes AQP4 polarization as a key modulator of this dysfunction, showing partial recovery in peri-infarct transition zones chronically, thereby identifying therapeutic targets and windows.

In recent years, various imaging methods have been developed to visualize the glymphatic pathway. After injecting fluorescent tracer into the cerebrospinal fluid at cisterna magna, one method is *ex vivo* imaging of micron-thick brain sections used to evaluate the distribution and deposition in the whole brain. Another method is *in vivo* two-photon imaging used to visualize the movement of the fluorescence tracer (Iliff et al., 2012; Pizzo et al., 2018; Iliff et al., 2014). In clinical practice, a gadolinium-based contrast agent was intrathecally injected to mimic the imaging of a fluorescent tracer, with dynamic T1-weighted imaging performed to dynamically track the cerebrospinal fluid flow in and out. To date, this method is limited to patients with certain diseases who need lumbar puncture and cannot be routinely carried out in clinical practice. DTI-ALPS, as a non-invasive method, has been developed in recent years. Previous study has confirmed the consistency between DTI-ALPS and dynamic enhanced MRI with intrathecal injection of the gadolinium contrast agent (Tavazzi et al., 2022). Our study further detailed the dynamic changes of the ALPS index and illuminated its correlation with AQP4 polarization.

To the best of our knowledge, this is the first animal study tracking glymphatic dynamics via DTI-ALPS. Key advantages include longitudinal ALPS monitoring from hyperacute to chronic phases and correlating ALPS with AQP4 polarization, which is unfeasible in human studies. We also proposed the corpus callosum/cingulate gyrus as a novel ROI, expanding measurement options. Furthermore, we stratified peri-infarct regions into glial scar and transition zones based on astrocyte/AQP4 spatiotemporal features, demonstrating matched AQP4 polarization and ALPS index dynamics.

Despite these findings, unresolved questions persist. The glymphatic function is globally impaired under the ischemic insult, and the mechanism remains unknown. In the previous study, this phenomenon was attributed to the widespread changes in glial water transport. However, in our study, there were no significant changes in astrocytes and AQP4 in the contralateral hemisphere. Considering the wide network of the glymphatic pathway, we surmised that waste solutes of the infarct core could also flow into the contralateral side and influence the contralateral glymphatic function. Additionally, systemic factors, such as neuroinflammation or blood–brain barrier (BBB) disruption triggered by the ischemic injury, might also contribute to the contralateral glymphatic impairment despite minimal local AQP4 changes. This speculation needs further study. The present study had certain limitations: (1) Anesthesia may have confounded glymphatic flow despite standardized protocols. (2) Manual ROI tracing, especially in lesioned areas, risks subjective bias despite randomization and anatomical landmarks. (3) The lack of behavioral data collection limits the assessment of functional correlates with the observed imaging and pathological changes.

## Conclusion

The ALPS index decreased in the hyperacute stage and recovered in the early chronic stage of cerebral ischemia. The ALPS index reflects AQP4 polarization status changes in the transition area around the infarction.

## Data Availability

The original contributions presented in the study are included in the article/supplementary material, further inquiries can be directed to the corresponding author/s.
